# S24. IS IT FEASIBLE TO PREDICT LONG-TERM METABOLIC OUTCOMES IN PSYCHOSIS USING BIOLOGICAL PROFILING AT BASELINE?

**DOI:** 10.1093/schbul/sby018.811

**Published:** 2018-04-01

**Authors:** Conrad Iyegbe, Lauren Allen, John Lally, Marta DiForti, Robin Murray, Fiona Gaughran

**Affiliations:** 1 Department of Psychological Medicine; 2 Department of Psychosis Studies; 3 Centre for Social, Genetic and Developmental Psychiatry

## Abstract

**Background:**

Antipsychotic medications are widely prescribed for the treatment of psychotic disorders but carry a variable propensity to increase weight. Thus metabolic dysfunction is the primary cause of premature death in psychosis patients. A system-based approach to understanding the molecular mechanisms behind metabolic dysfunction can potentially provide scope for tailored interventions and alternative treatment pathways that avert such risks on an individual basis. The aim of this study is to identify transcriptomic predictors of high Body Mass Index (BMI) and blood glucose in first episode and chronic psychosis patients.

**Methods:**

100 first-episode and 100 chronic cases of psychosis meeting ICD-10 criteria (F20-29 and F30-33) were recruited as part of 2 independent studies from 3 NHS Trusts: South London and Maudsley (SLAM), Oxleas and Sussex. Cases were ethnically mixed and aged between 18–65. All participants gave informed consent for biological sampling and a range of physical health assessments. Blood glucose was measure using HbA1c while height and weight data were also taken and used to calculate BMI. For FEP subjects biological measures were taken at baseline, 3 months and 12 months post recruitment. RNA samples were collected at the baseline timepoint via PAXgene blood tubes and interrogates, using the Illumina HumanHT-12.v4 beadchip array. Samples were run at the National Institute for Health Research’s (NIHR) Biomedical Research Centre for Mental Health (BRC-MH) at the Institute of Psychiatry, Psychology and Neuroscience. A total of 4756 probes passed a stringent quality control across the 200 samples.

**Results:**

Quantitative data on BMI and hba1c levels were used to assess the predictive efficacy of variables grouped by source (ie. clinical, demographic, technical and transcriptomic features) in first episode psychosis patients. All the predictor categories were included in the initial model, although individual categories were then dropped one at a time. This leave-one-out strategy allowed the direction, impact and relative contribution of the different feature classes to be assessed. Gene-expression and clinical features were consistently associated with the lowest Mean Squared Error after 100 iterations of K-fold cross-validation and after 11 different values of the alpha parameter across 500 imputed datasets. Hba1c or BMI was used as the clinical predictor, depending on whether Hba1c or BMI was used as the target variable. Unattributed surrogate variables derived from surrogate variable analysis (n=6) were analysed within the technical feature set. Having established that gene expression has inherent value as a predictor of metabolic status the same analytical steps were repeated for the discretised versions of these traits (ie. diabetes and obesity). Top-ranking gene transcripts were compared between the quantitative and discretised models. Rank lists of transcripts were subsetted to allow the power distribution across ordered transcripts to be profiled.

**Discussion:**

The top performing transcripts identified are undergoing validation analysis in the chronic sample. Results will be conveyed in terms of sensitivity and false positive rates (ie. the area under the Receiver Operating Characteristic curve). We will undertake further validation through trajectory analysis of gene-expression profiles in followed-up patients.

